# Suicidal ideation during the first wave of COVID-19 pandemic in a child and adolescent psychiatry emergency care sample

**DOI:** 10.1192/j.eurpsy.2021.1697

**Published:** 2021-08-13

**Authors:** F. Cordeiro, V. Santos, T. Cartaxo

**Affiliations:** Child And Adolescent Psychiatry, Pediatric Hospital of Coimbra, Coimbra Hospital and Universitary Centre, Coimbra, Portugal

**Keywords:** Restrictions, pandemic, COVID-19, Suicidal ideation

## Abstract

**Introduction:**

The COVID-19 pandemic forced many countries to apply restrictive measures. During the first wave Portugal went through a lockdown, and all the child and adolescents had to stay home and could only contact with the one’s they lived with for several months.

**Objectives:**

This study aimed to evaluate the impact of those restrictions on suicidal ideation in the pediatric population evaluated in a child and adolescent psychiatry emergency care of a tertiary referral hospital.

**Methods:**

We conducted an exploratory retrospective study. All the data from discharge notes were collected between March 15^th^ and June 15^th^ of 2020 (n=59), and in the homologous period of the previous year (n=178). The referral after evaluation (primary care, child and adolescent psychiatry consultation, inpatient unit) was considered a measure of severity.

**Results:**

The demographic variables (sex, age) were homogeneous between the two groups (p ≥ 0,05). 17,4% (n=31) of the sample from 2019, and 16,9% (n=10) of the sample of 2020 had suicidal ideation, which was not statistically different between groups (p=1,000). The referral, after evaluation between groups were also not statistically different (p=0,186).

**Conclusions:**

Even though the proportion of patients with suicidal ideation was homogenous during the two periods, the total number of patients evaluated in the emergency room were lower during the first wave of Covid-19 pandemic. We assume that the population had fear of seeking help in hospital facilities, but we also believe that the pause on school burdens and the reconnection between some families could have function as protective factors.

**Disclosure:**

No significant relationships.

